# MicroRNA Expression is Associated with Sepsis Disorders in Critically Ill Polytrauma Patients

**DOI:** 10.3390/cells7120271

**Published:** 2018-12-13

**Authors:** Alexandru Florin Rogobete, Dorel Sandesc, Ovidiu Horea Bedreag, Marius Papurica, Sonia Elena Popovici, Tiberiu Bratu, Calin Marius Popoiu, Razvan Nitu, Tiberiu Dragomir, Hazzaa I. M. AAbed, Mihaela Viviana Ivan

**Affiliations:** 1 Faculty of Medicine, “Victor Babes” University of Medicine and Pharmacy, 300041 Timisoara, Romania; alexandru.rogobete@umft.ro (A.F.R.); dsandesc@yahoo.com (D.S.); bedreag.ovidiu@umft.ro (O.H.B.); marius.papurica@umft.ro (M.P.); popovici.sonia@yahoo.com (S.E.P.); tiberiu.bratu@umft.ro (T.B.); razvan.nitu@umft.ro (R.N.); tiberiu.dragomir@umft.ro (T.D.); hazzaa.aabed@gmail.com (H.I.M.A.); viviana.ivan@umft.ro (M.V.I.); 2 Clinic of Anesthesia and Intensive Care, Emergency County Hospital “Pius Brinzeu”, 300723 Timisoara, Romania

**Keywords:** microRNAs, epigenetic biomarker, sepsis, inflammation

## Abstract

A critically ill polytrauma patient is one of the most complex cases to be admitted to the intensive care unit, due to both the primary traumatic complications and the secondary post-traumatic interactions. From a molecular, genetic, and epigenetic point of view, numerous biochemical interactions are responsible for the deterioration of the clinical status of a patient, and increased mortality rates. From a molecular viewpoint, microRNAs are one of the most complex macromolecular systems due to the numerous modular reactions and interactions that they are involved in. Regarding the expression and activity of microRNAs in sepsis, their usefulness has reached new levels of significance. MicroRNAs can be used both as an early biomarker for sepsis, and as a therapeutic target because of their ability to block the complex reactions involved in the initiation, maintenance, and augmentation of the clinical status.

## 1. Introduction

Critically ill polytrauma patients present one of the most complex clinical pictures that the intensivist and trauma team will encounter in their careers [1,2,3,4,5]. The complexity of these cases is due both to the initial traumatic injury, and to the secondary post-traumatic responses to injury [2,3,4,5,6,7,8,9,10,11,12,13,14,15,16]. Moreover, through the interactions of molecular mechanisms with other, initially functional systems, and through molecular denaturation reactions, the critically ill polytrauma patient becomes a complex medical case from a clinical and molecular point of view. A series of complex mechanisms involved in the pathophysiology and biochemistry of sepsis have been studied for the past several years. However, the critically ill polytrauma patient is so complex biochemically and molecularly that no specific biochemical pathways have been found in which intervention could increase survival rates, or decrease the incidence of sepsis and multiple organ dysfunction syndrome (MODS) [3,4,5,6,7,8,9,10,11,12,13,14,15,16,17,18,19,20,21,22,23,24,25]. However, in the last few years, a series of microRNA epigenetic species have been identified. These species are responsible for the modulation of certain complex molecular reactions. Furthermore, numerous studies have shown the importance of microRNAs in early diagnosis and possible future epigenetic therapies [6,7,8,9,10,11,12,13,14,15,16,17,18,19,20,21,22,23,24,25,26,27,28,29,30,31,32,33]. By examining microRNAs with respect to critically ill polytrauma patients, we can see important links between the development and modulation of the systemic inflammatory response, the immune system, coagulation status, and response to infections [7,8,9,10,11,12,13,14,15,16,17,18,19,20,21,22]. The paper aims to systematize the microRNA expressions that are closely related to the pathophysiological events involved in a critically ill polytrauma patient with sepsis. Moreover, we wished to highlight the most important microRNA expression studies conducted to date that could be used as biomarkers for the early diagnosis of sepsis.

## 2. Biochemical and Biosynthesis Aspects of MicroRNAs

From a molecular point of view, microRNAs are synthesized in the cell nucleus through the action of RNA polymerase II on certain specific genes. Hence, the initial species, the pri-microRNAs, are formed following complex reactions [13,14,15,16,17,18,19,20,21,22,23,24,25,26,27,28,29,30,31,32,33,34,35]. In the next step, RNAse III endonuclease, also called Drosha, activates the pri-microRNAs. This reaction is catalyzed by the DiGeorge Syndrome Critical Region 8 (DGCR8) complex, which leads to the formation of pre-microRNAs [15,34,35,36,37,38,39,40,41,42,43,44,45,46,47,48,49,50,51,52,53,54,55,56,57,58,59,60,61,62,63,64,65,66,67,68,69,70,71,72,73,74].

**Figure 1:**
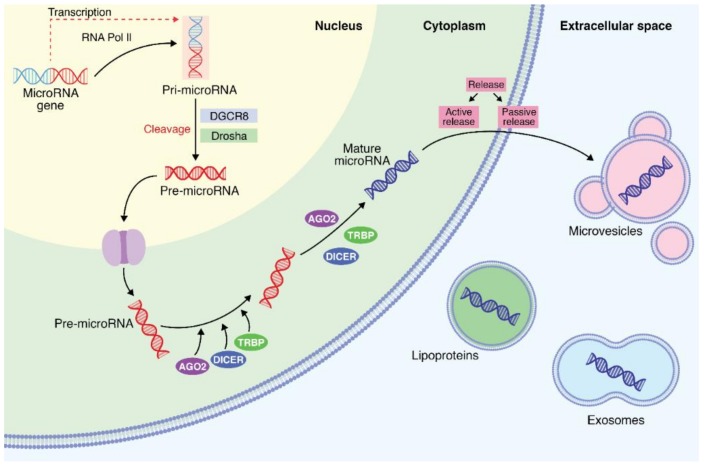
MicroRNA biosynthesis mechanisms. For further explanation, please see the details in the text. RNA pol II—RNA polymerase II; pri-microRNA—primitive microRNA; DGCR8—DiGeorge Syndrome Critical Region 8; Drosha—RNAse III endonuclease; pre-microRNA—precursor of microRNA; AGO2—endonuclease Argonaute 2; Dicer—Rnase III endonuclease; TRBP—transactivation response element RNA-binding protein.

Once these almost final species are formed, the pre-microRNAs bind with the Exportin-5 transporter protein, which shifts them from the nucleus into the cytoplasm. Inside the cytoplasm, a new reaction, initiated by an RNAse III endonuclease called Dicer and by the RNA binding protein (TRBP), takes place, which leads to the formation of the final microRNA species [17,20,66,67,68,69,70,71,72,73,74,75,76,77,78,79,80,81,82,83,84,85,86,87,88]. The last step involves coupling the RNA-induced silencing complex (RISC) [18,78,79,80,81,82,83,84,85,86,87,88,89,90,91]. The final molecular species is then transported outside the cell through different mechanisms and in various forms, such as ribonucleoprotein complexes, microvesicles, exosomes, and high-density lipoproteins (Figure 1) [14].

## 3. MicroRNA Identification from Different Body Fluids

**Figure 2:**
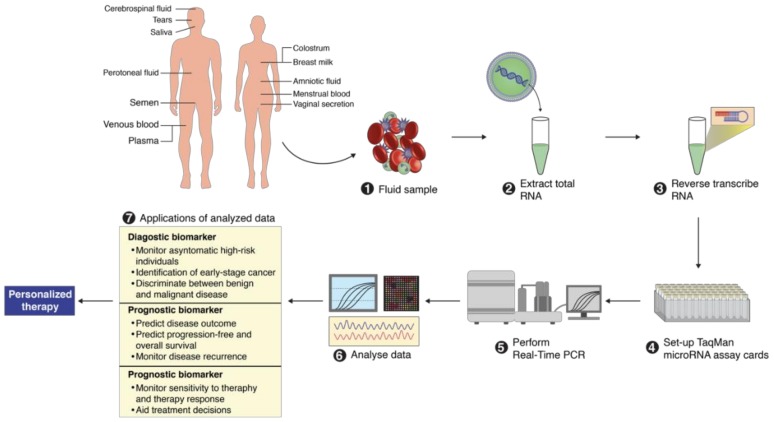
MicroRNA identification workflow from different body fluids.

MicroRNAs have been proposed as possible biomarkers because of the research evidence that shows that changes in a range of cellular microRNAs correlate with various pathophysiological conditions, including inflammation, oxidative stress, sepsis diabetes and different types of cancer [33,34,35,36,37,38,39,40,41,42,43,44,45,73,74,75,76,77,78,79,80,81,82,83,84,85,86,87,88,89,90,91,92,93]. These molecules have also been known for their low complexity, simple detection and amplification, tissue-restricted expression profiles, and sequence conservation between human and model organisms [94,95,96,97,98]. However, they have not been incorporated into clinical practice due to several factors such as the lack of a universal and comprehensive measurement technique that would be convenient enough in terms of handling, the rate of analysis, and reliability [96,97,98,99,100,101]. Apart from the measurement technique, another factor that has been holding back the use of microRNAs is that their concentration in the body is relatively low. However, there exist measurement methods that have been routinely used, although they have their advantages and disadvantages. These techniques include small RNA sequencing, quantitative reverse transcription polymerase chain reaction (qPCR), and microarray hybridization. All of these are applied according to the respective propose of analysis (Figure 2). When it comes to the successful identification of these microRNAs, the factors that are critical, such as the choice of the measurement sample and the appropriate normalization strategy come into play. The profiles of these important biomarkers are also influenced considerably by exogenous factors such as medication, nutrition, and certain environmental conditions [98,99,100,101,102,103,104,105,106,107,108,109,110,111,112,113,114].

## 4. Importance of MicroRNAs for Clinical Use

Due to their unique features, such as disease specificity, relative stability, and easy accessibility, microRNAs are considered the future biomarkers for the diagnosis and prognosis of specific diseases as well as monitoring therapeutic responses in clinical settings [75,76,77,78,79]. MicroRNAs have been identified in different clinical settings, and their importance as biomarkers is still under investigation [80,81,82,83,84,85,86,87,88,89,90,91,92,93,94,95,96,97,98,99,100,101,102,103,104,105,106,107,108,109,110,111,112,113,114,115,116,117]. For example, a number of these molecules have been associated with sepsis, acute lung injury and acute organ dysfunction diagnoses. MicroRNAs are also being considered for therapeutic purposes where up-regulatory or down-regulatory molecules, targeting specific microRNAs, can be administered with the aim of managing specific pathological conditions. A study of this is currently at the clinical trial stage. Studies have shown that the anesthetics and medications used in post-operative patient care affect the expression of microRNA, which in turn affects the functioning or survival of certain types of cells in the body, such as neurocytes. The expression of microRNAs in their various cells are highly specific, and therefore, they have a distinct display pattern in different tissues, which contributes to their characteristic features and functions. With this in mind, these molecules have been used to detect the presence of disease or tissue malfunction due to their recognizable pattern of appearance [97,98,99,100,101,102,103,104,105]. For instance, rough relations have been created linking specific microRNA expressions to the manifestation of certain pathological conditions, including microR-21 being shown as a proto-oncogene in adenocarcinoma, and microR-146a acting as an inhibitory factor to inflammatory processes by dampening the nuclear factor-kB (NF-kB) signaling [114,115,116]. MicroRNA has also been considered in forensic investigations due to their initially-named properties. It helps address the challenge of sensitivity and specificity when it comes to criminal identification. However, this is entirely dependent on the method applied in such endeavors. In criminal investigations, the disadvantages of microRNA profiling have not yet been studied [116,117].

## 5. Roles of MicroRNAs in the Pathophysiology of Oxidative Stress Associated with Sepsis

**Figure 3:**
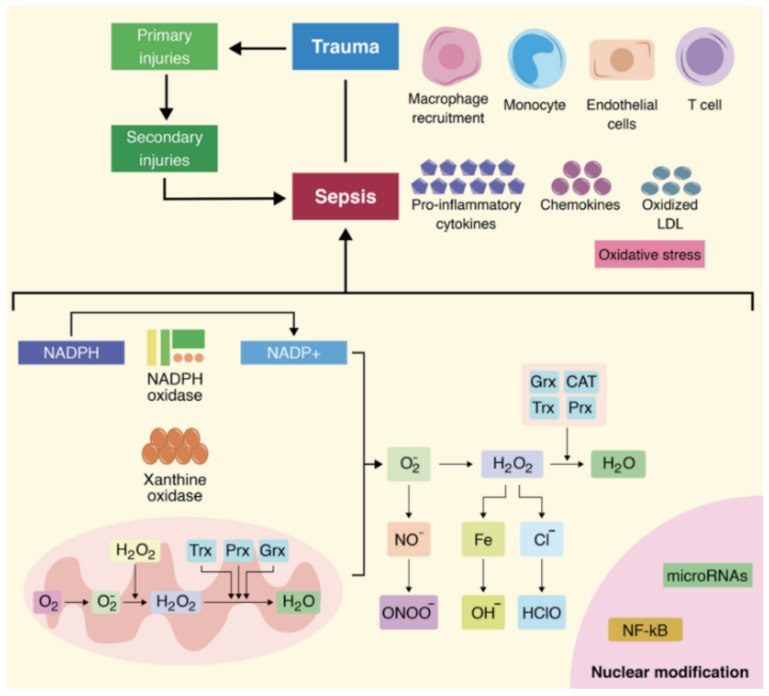
The critically ill polytrauma patient is characterized by a series of secondary, post-traumatic injuries, represented especially by cellular and molecular damage. Oxidative stress is an important molecular phenomenon, and it has important links with a series of bio-macromolecular systems. An important source of free radicals is the mitochondria, where huge amounts of free oxygen radicals are produced that will further lead to the augmentation of the pro-oxidative phenomena. Moreover, the molecular disaster will continue as other systems are affected such as the endovascular system, lipid molecules, proteins, and cellular organelles.

Under normal conditions, the human body synthesizes numerous biochemical species with increased reactivity compared to the existent macromolecules. These species are called free radicals and are divided depending on their origin into reactive oxygen species, reactive lipid species, reactive nitrogen species, and other more complex redox systems [20,21,22,23,24,25,26,27,28,29,30,31,32,33,34,35,36,37,38,39,40,41,42,43,44,45,46,47,48,49,50,51,52,53,54,55,56,57,58,59,60,61,62,63,64,65,66,67,68,69,70,71,72,73,74]. Biochemically speaking, the most aggressive free radicals are oxygen radicals such as hydroxyl radicals (HO^−^), superoxide anions (O_2_^−^), or hydrogen peroxide (H_2_O_2_). Moreover, nitrogen radicals such as peroxynitrite (ONOO^−^) and nitric oxide (NO), or the lipid radicals, especially the lipid peroxyl (LOO^−^), also present similarly high reactivity [3,19,20]. The oxidative stress appears once the free radicals accumulate over the endogenous antioxidants. Under circumstances of traumatic stress, a series of endogenous systems are responsible for generating an excessive amount of free radicals. Among these, the most studied are mitochondrial respiration, the xanthine reduction mechanisms, and the NADPH oxidase enzymatic system [15,21,22,23,24]. Admittedly, under physiological conditions, the human body has a series of biomacromolecules with antioxidant capacities such as catalase (CAT) [25], superoxide dismutase (SOD) [26], peroxiredoxins (PRXs) [27], glutathione (GSH) [28], and glutaredoxins (GRXs). However, in the case of critically ill polytrauma patients, the production of free radicals overcomes the endogenous antioxidant capacity of the body, and therefore oxidative stress associated with the systemic inflammatory response appears very quickly. In the case of critically ill polytrauma patients, the pro-oxidative phenomenon appears at the moment of trauma because of the associated organic injuries. A short time after the traumatic impact, the molecular injury will be transmitted, augmented, and multiplied in the cell, especially inside the cellular organelles [29,30,31]. From a clinical point of view, the molecular lesions have important implications in the clinical evolution of a patient due to the significant increase in the morbidity and mortality rates through their association with the systemic inflammatory response, and also because of their association with generalized infections [32,33,34,35,36]. With regard to the association with infections, oxidative stress has significant implications in increasing the incidence of sepsis due to the release of free radicals, cytokines, and adhesion molecules. Hence, immunosuppression, the increased concentration of pro-inflammatory factors, and the aggressive attack of free radicals all lead to MODS in the critically ill polytrauma patient, despite complex treatment options (Figure 3) [37,38,39,40,41,42,43,44,45,46,47,48,49,50,51,52,53,54,55,56,57,58,59,60,61,62,63,64,65,66,67,68,69,70,71,72,73,74,75,76,77,78,79,80,81,82,83,84,85].

The microRNA species play an important role in the propagation of pro-oxidative signals by modifying the reactivity of the molecular receptors. Numerous studies have identified important implications of microRNAs for the cis-acting DNA sequences [41,42,43]. Practically, the cellular proliferation is influenced by microRNA-9 by modulating the activity of orphan nuclear receptor TLX, located in the neuroepithelium. Another example is the estrogen and androgen receptors that have microRNA-21, microRNA-222, microRNA-221, microRNA-101, microRNA-206, microRNA-433, microRNA-34a, microRNA-125b, and microRNA-127 as genetic substrates [44,45].

The nuclear transcription factor kB (NF-kB) represents another interesting aspect of a molecular attack. From a biochemical viewpoint, NF-kB is involved in modifying the reactions of certain genes and is influenced in most cases by a series of external or internal factors such as the IKB and IKK proteins [9,46,47]. If we were to discuss the links between NF-kB and oxidative stress, and the implications of NF-kB in the clinical outcome of these patients, one could highlight the cellular adaptability induced by the pathophysiological changes arising from inflammation, infections, and the immune response. This can be explained through the implications that NF-kB has in the production of pro- and anti-inflammatory cytokines such as interleukin-1 (IL-1) and tumor necrosis factor-alpha (TNF-alpha) [48,49,50,51]. Moreover, in this complex series of events that make up a molecular disaster, there are numerous important links caused by the reciprocal activation of certain factors that are decisive in the augmentation of the molecular disaster. In the case of critically ill patients, a series of specific secondary phenomena occur, such as tissue hypoxia, generalized inflammation, and infections [52]. With regard to this, researchers have identified the microRNAs that play a decisive role in the modification of the biochemical pathways. An important study carried out by Scott et al. reported significant changes in the expression of microRNA-17-92, microRNA-221, microRNA-126, and microRNA-222 [53]. In the literature, other microRNA species that have important implications for endothelial damage have also been reported, such as microRNA-278 and microRNA-146 [54]. Another study carried out by Kung et al. reported reduced activity for microRNA-26a, microRNA-126, and microRNA-24 [55]. The same study showed increased expression of microRNA-346, microRNA-30b, microRNA-999, and microRNA-30a.

With regard to epigenetic expression in tissue hypoxia, microRNAs have been shown to have multiple implications, both augmenting cellular destruction and increasing the pro-inflammatory and pro-oxidative status [80,81,82,83,84]. Numerous microRNAs are responsible for dictating the biosynthesis for adhesion molecules, free oxygen, nitrogen, or lipid radicals, and affecting cell and mitochondria energy. Among these, the most microRNAs that have been most studied in-depth are microRNA-213, microRNA-210, microRNA-24, microRNA-27, microRNA-23, microRNA-26, microRNA-210-3p, microRNA 23b-3p, microRNA-1275, microRNA-210-3p, microRNA-145-5p, microRNA-92b-3p, microRNA-181a-2-3p, microRNA-185-5p, microrRNA-20a-5p, and microRNA-92b-3p [84,85,86,87]. Another associated phenomenon is ischemia–reperfusion syndrome. From a clinical and molecular point of view, ischemia–reperfusion is an important generator of free radicals and inflammatory molecules that are responsible for aggravating the clinical status of these patients, especially in the context of inflammation and infection. Important changes in epigenetic expression have also been identified in the case of ischemia–reperfusion syndrome. Among these, the most commonly studied are microRNA-290, microRNA-26, microRNA-192, microRNA-805, microRNA-194, microRNA-187, microRNA-145, and microRNA-21 [14,88,89].

**Figure 4:**
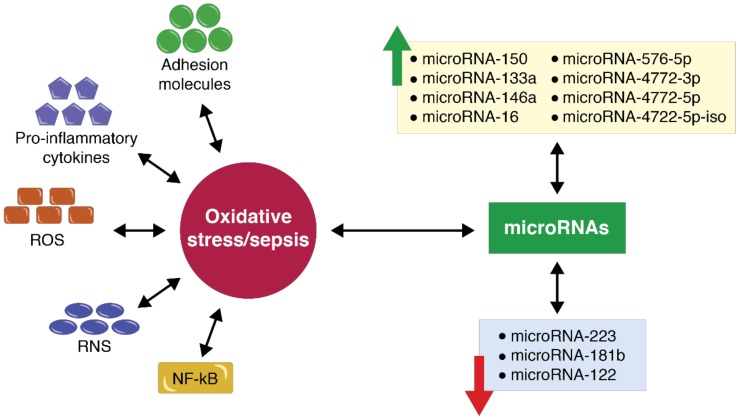
MicroRNA expression in a critically ill polytrauma patient with sepsis. A short time after the primary traumatic injury, the critically ill polytrauma patient develops a series of secondary post-traumatic injuries, especially molecular and cellular injuries. Among these, the most studied are the excess biosynthesis of free radicals reactive oxygen species (ROS) and reactive nitrogen species (RNS), and the augmentation of the pro-oxidative chain. Moreover, together with the involvement of the immune system, the activation of nuclear transcription factor kappa B (NF-kB), the emergence of adhesion molecules, the release of excess pro-inflammatory factors, and infections will determine a series of microRNA species that will intervene in the modulation of this complex molecular cycle. Numerous studies have shown both an increase in the activity of certain microRNA species, and a decrease in the expression of other species in certain selected cases [14,80,81,82,83].

A high proportion of critically ill polytrauma patients develop acute respiratory distress syndrome (ARDS). From a cellular and molecular viewpoint, in ARDS, the neutrophils invade the pulmonary tissue leading to the initiation of aggressive pro-inflammatory mechanisms [56,48,70]. The molecular cascade in this case is activated and augmented by the excess production of interleukin 6 (IL-6), interleukin 1 beta (IL-1), and tumor necrosis factor alpha (TNF-alpha). Furthermore, this molecular cascade leads to increased vascular permeability in the pulmonary tissue. The molecular reactions are extremely complex, with the inhibition of apoptosis in the alveolar capillaries through the action of vascular endothelial growth factor (VEGF) [60,71,72]. The VEGF receptors, including vascular endothelial growth factor receptor 1 (VEGFR1) and vascular endothelial growth factor receptor 2 (VEGFR2) are further activated, leading to increased vascular permeability. In this case, the expression of microRNAs also plays an important role, modulating a series of complex molecular reactions [73,74]. Yehya et al. reported important changes for microRNA-466c-5p, microRNA-466d-5p, microRNA-15b, microRNA-154, microRNA-466c, microRNA-466b, microRNA-466f-3p, microRNA-375, microRNA-378, microRNA-347, and microRNA-32* [75]. A similar study carried out on the same group of patients by Kulshreshtha et al. reported changes in the expressions of miRNA-27, miRNA-103, miRNA-107, miRNA-26, miRNA-181, miRNA-210, miRNA-23, miRNA-24, and miRNA-213 [76]. Likewise, important changes have been noted in these situations for miRNA-194, miRNA-214, miRNA-223, miRNA-100, miRNA-140, miRNA-142-3p, miRNA-25, miRNA-27b, miRNA-181c, miRNA-21, and microRNA-224 activity [77]. Tacke et al. reported an increased expression for microRNA-133a in patients with sepsis [78]. Wang et al. also reported the decreased expression of microRNA-223, microRNA-181b, and microRNA-146a [79] (Figure 4).

Hyperoxia is closely related to ARDS. This phenomenon is mostly induced by the intensive care physician because of difficult ventilation and inadequate oxygen concentrations in the circulatory system. In these situations, the intensive care unit (ICU) Fi-inspired oxygen fraction (FiO_2_) is usually increased to 1.0 (100% O_2_). On the other hand, increasing FiO_2_ to 1.0 directly affects the mitochondria and the microvascular system. Together with the impairment of the microvascular system, the vascular perfusion in the pulmonary tissue will drop significantly, leading to a decreased gas-exchange capacity and the progressive deterioration of the patient’s clinical status.

Vascular endothelial growth factor (VEGF) has been widely discussed in the literature in relation to microvascular injury. Specifically, a series of reactions involved in the inhibition of apoptosis in the alveolar capillaries has been mentioned in the literature. Moreover, an important increase in the expression of vascular endothelial growth factor receptor 1 (VEGFR1) in patients with ARDS has been demonstrated [58,59,60].

There are complex mechanisms that are closely related to the biofunctionality of the endothelial surface. An important system in this category is the KL-6 glycoprotein that can be found on the surface of type II alveolar cells [61]. From an immunological point of view, T-cell expression is widely influenced by all of these mechanisms. Recent studies have shown changes in the Foxp3+ regulatory T-cell (Tregs), CD4+, CD3+, CD25hi, CD127lo, and CD25+ expression [62].

## 6. MicroRNA Expression in the Case of Polytrauma Patients with Sepsis

From a pathophysiological and molecular viewpoint, in the case of polytrauma patients with sepsis, an important phenomenon appears due to excess cytokine synthesis. This is determined by the complex reactions between the lipopolysaccharide macromolecules (LPS) and lipopolysaccharide binding protein (LPB) [63,64]. The molecular bond required for these reactions to take place exists due to the CD14 receptor found on the surface of macrophages. Apart from these complex links, there are a series of other reactions represented especially by the synthesis of certain mediators, such as histamines, chemokines, or different hormones. Moreover, the coagulation cascade plays an important role in the augmentation and self-propagation of the molecular phenomena involved in sepsis. The most important pro-inflammatory and anti-inflammatory mediators are interleukin 4 (IL-4), interleukin 10 (IL-10), interleukin 17 (IL-17), interleukin 1 (IL-1), interleukin 2 (IL-2), interleukin 6 (IL-6), interleukin 12 (IL-12), interleukin 8 (IL-8), Procalcitonin (PCT), N-terminal C natriuretic peptide (NT-CNP), C-reactive proteins (CRP), tumor necrosis factor alpha (TNF-alpha), interferon gamma (INF-gamma), and transforming growth factor beta (TGF-beta) [14,49,65,66].

Recent studies have shown a series of implications for microRNA species in the pathophysiology of sepsis, pro-inflammatory and pro-oxidative phenomena (Table 1). MicroRNA-146a, microRNA-150, and microRNA-233 have complex implications in the molecular damage in sepsis [67]. Moreover, Puskarich et al. reported strong statistical correlations between the changes in microRNA-150 expression and the increase in mortality rates. Another important study, carried out by Vasilescu et al. reported decreased activity for microRNA-150 and microRNA-342-5p in the case of septic patients. On the other hand, there was an increased expression of microRNA-486 and microRNA-182 in these patients [67]. A similar study carried out by Benz et al., identified important changes in microRNA-233 in patients with sepsis [68]. Other species involved in the molecular and genetic sepsis reactions include microRNA-340, microRNA-324-3p, microRNA-16, microRNA-210, let-7b, microRNA-15b, microRNA-484, microRNA-486-5p, and microRNA-324-3p [69].

Moreover, numerous studies have reported a series of microRNAs that did not show significant changes regarding their expression in sepsis. Such microRNA species have been identified both in the patients’ serum (microRNA-451, [95], microRNA-494 [90]), as well as in the plasma (let-7i [90]) and blood (microRNA-21, microRNA-503, microRNA-155, microRNA-486-5p, microRNA-132, microRNA-203, and microRNA-1249 [90,91,92,93,94,95]) (Table 2).

## 7. Conclusions

The complexity of the pathophysiological and molecular mechanisms in critically ill polytrauma patients is very high. They are responsible for the worsening of the patient’s clinical status under certain circumstances. Understanding and preventing certain biomolecular and epigenetic mechanisms could lead to decreased molecular and cellular injury, as well as a lower overall risk for these patients. MicroRNA expression is a strong candidate for the future of intensive care because of the early diagnosis opportunity and because of its capacity to interact with certain key points of the biochemical pathways. Among these, the most widely-studied species are represented by microRNA-150, microRNA-133a, microRNA-146a, microRNA-576-5p, microRNA-4772-3p, microRNA4772-5p, and microRNA4722-5p-iso—the expression of which is highly augmented—as well as microRNA-223, microRNA-181b, and microRNA-122, which have lower levels in sepsis patients. Moreover, these microRNA species can be determined in different body fluids, such as serum, plasma, and blood, widening the range of options for the epigenetic determination of sepsis in critically ill polytrauma patients. However, until now, the epigenetic interactions in a clinical context have not been clearly reported, and further studies are necessary to identify the correct context for microRNA expression.

## Figures and Tables

**Table 1:** MicroRNA expression in sepsis.

Involved MicroRNAs	Body Fluid of Identification	Expression	References
microRNA-4772-5p	Serum	Up-regulated	[90,91,92]
microRNA-4772-5p Iso	Serum	Up-regulated	[92]
microRNA-15a	Serum	Up-regulated	[90,91]
microRNA-16	Serum	Up-regulated	[90]
microRNA-574-5p	Serum	Up-regulated	[91]
microRNA-4772-3p	Serum	Up-regulated	[92]
microRNA-4516	Serum	Up-regulated	[93]
microRNA-454-3p	Serum	Up-regulated	[93]
miR-155-3p	Serum	Up-regulated	[93]
microRNA-219b	Serum	Up-regulated	[94]
microRNA-1889	Serum	Up-regulated	[94]
microRNA-106a	Serum	Up-regulated	[94]
microRNA-106b	Serum	Up-regulated	[94]
microRNA-205	Serum	Up-regulated	[94]
microRNA-20a	Serum	Up-regulated	[94]
miR-150	Serum	Up-regulated	[91]
microRNA-27a	Serum	Up-regulated	[95]
microRNA-122	Serum	Up-regulated	[96]
microRNA-146a	Serum	Up-regulated	[91]
microRNA-422	Serum	Up-regulated	[91]
microRNA-133a	Serum	Up-regulated	[90,91,92,93,94,95]
microRNA-4532	Serum	Up-regulated	[95]
microRNA-576-5p	Serum	Up-regulated	[80,81,82,83]
microRNA-483-5p	Serum	Down-regulated	[91]
microRNA-499-5p	Serum	Down-regulated	[91]
microRNA-193b*	Serum	Down-regulated	[91]
miR-146a-5p	Serum	Down-regulated	[93]
Let-7g-5p	Serum	Down-regulated	[93]
microRNA-30	Serum	Down-regulated	[94]
microRNA-199a-3p	Serum	Down-regulated	[93]
microRNA-29	Serum	Down-regulated	[95,96]
microRNA-297	Serum	Down-regulated	[96]
microRNA-125	Serum	Down-regulated	[96]
microRNA-25	Serum	Down-regulated	[96]
microRNA-19	Serum	Down-regulated	[95]
microRNA-182	Blood	Up-regulated	[95,96]
microRNA-15b	Blood	Up-regulated	[95]
microRNA-486	Blood	Up-regulated	[94,95,96]
microRNA-25	Blood	Down-regulated	[90]
microRNA-223	Blood	Down-regulated	[92,93,94,95]
microRNA-181b	Blood	Down-regulated	[90]
microRNA-342-5p	Blood	Down-regulated	[94,95]
microRNA-126	Blood	Down-regulated	[90]
microRNA-499-5p	Blood	Down-regulated	[90]

**Table 2:** MicroRNA expression unchanged in sepsis.

Involved MicroRNAs	Body Fluid of Identification	Expression	References
microRNA-451	Serum	Unchanged	[95]
microRNA-494	Serum	Unchanged	[90]
Let-7i	Plasma	Unchanged	[90]
microRNA-21	Blood	Unchanged	[90]
microRNA-503	Blood	Unchanged	[90]
microRNA-155	Blood	Unchanged	[91]
microRNA-486-5p	Blood	Unchanged	[95]
microRNA-132	Blood	Unchanged	[95]
microRNA-203	Blood	Unchanged	[90]
microRNA-1249	Blood	Unchanged	[90]
